# Supplementary figures and images for: Atypical structural tendencies among low-complexity domains in the Protein Data Bank proteome
Source: PLoS Comput Biol. 2020 Jan 27;16(1):e1007487. doi: 10.1371/journal.pcbi.1007487 (PMC7004392; doi:10.1371/journal.pcbi.1007487)

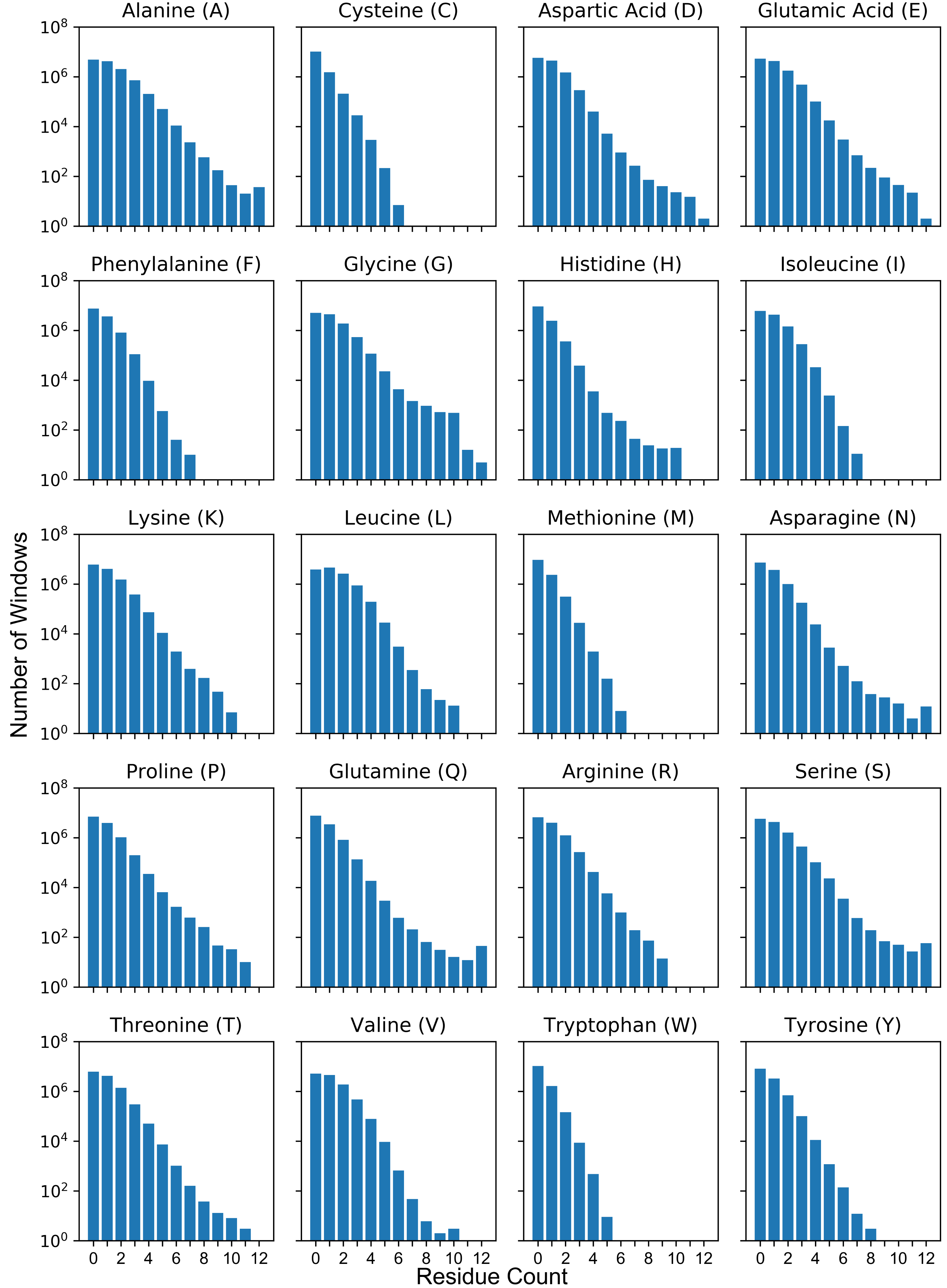

Supplement: S1 Fig — For each amino acid, the bar plot indicates the sample sizes in each residue count bin corresponding to Fig 4. (TIF) [file pcbi.1007487.s002.tif]

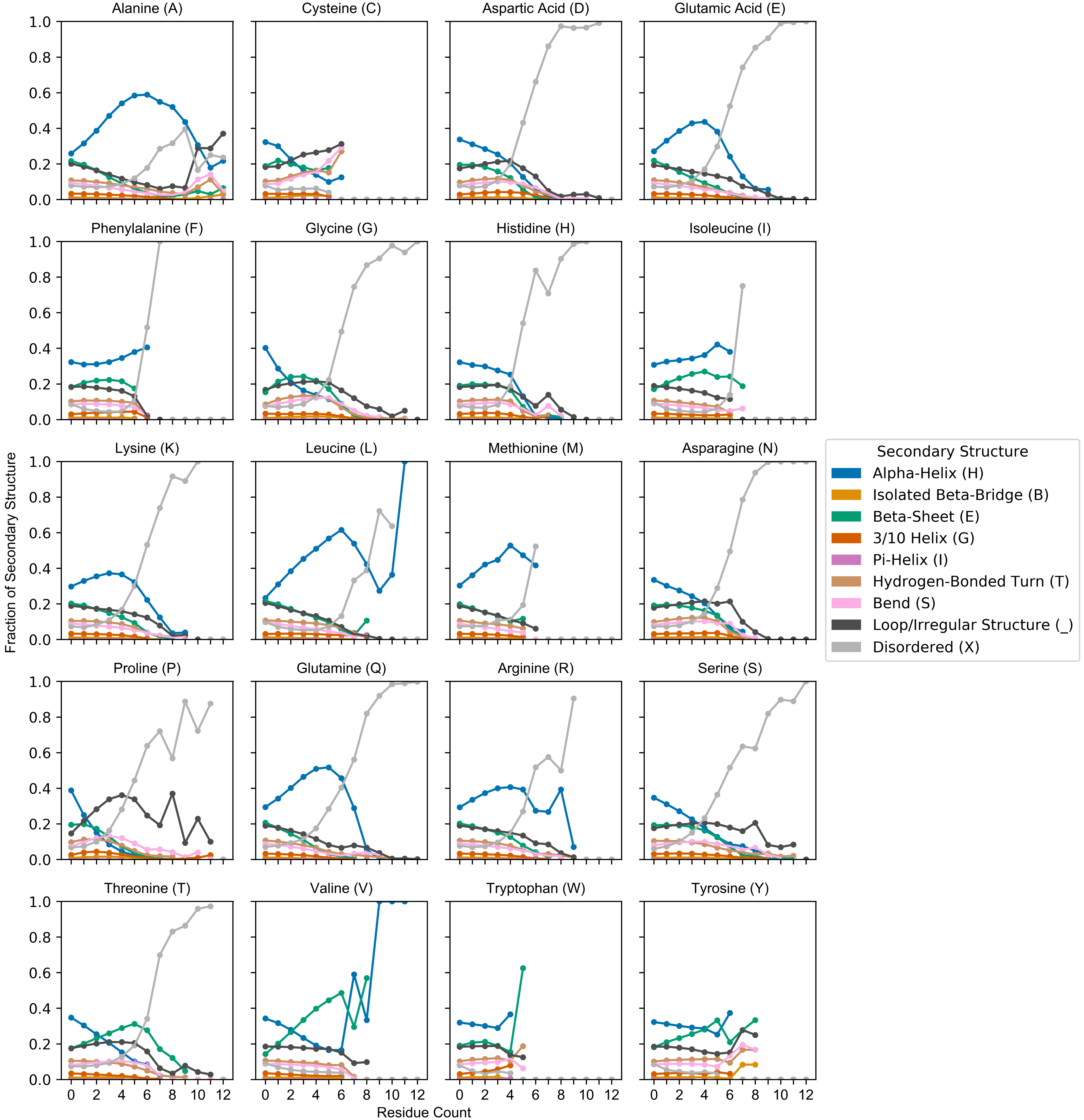

Supplement: S2 Fig — A set of PDB sequences with <40% sequence homology was analyzed with the same computational procedure (Fig 3) applied to the <90% homology PDB dataset. Compare with Fig 4 in the main text. It is worth noting that the PDB is updated regularly, and archived versions of the 40% non-redundant PDB are not available: therefore, the 40% non-redundant PDB proteome contains the structures (and sequences) of new proteins solved since our initial download of the 90% non-redundant PDB proteome. The full list of PDB chain IDs included in the 40% non-redundant PDB proteome is contained in S2 File. (TIF) [file pcbi.1007487.s003.tif]

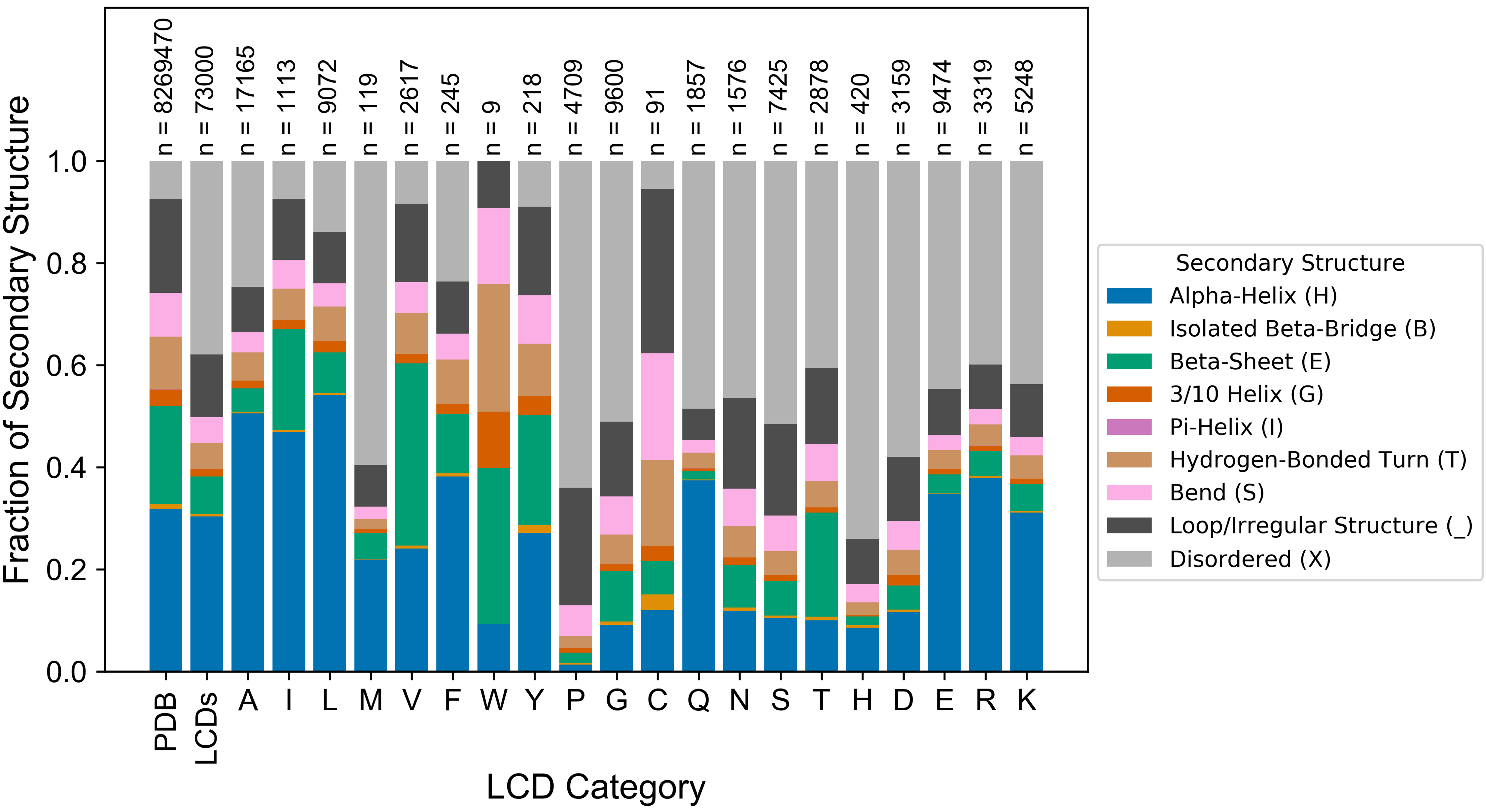

Supplement: S3 Fig — Classically-defined LCDs were identified among the 40% non-redundant PDB dataset in the same manner as described for Fig 5. (TIF) [file pcbi.1007487.s004.tif]

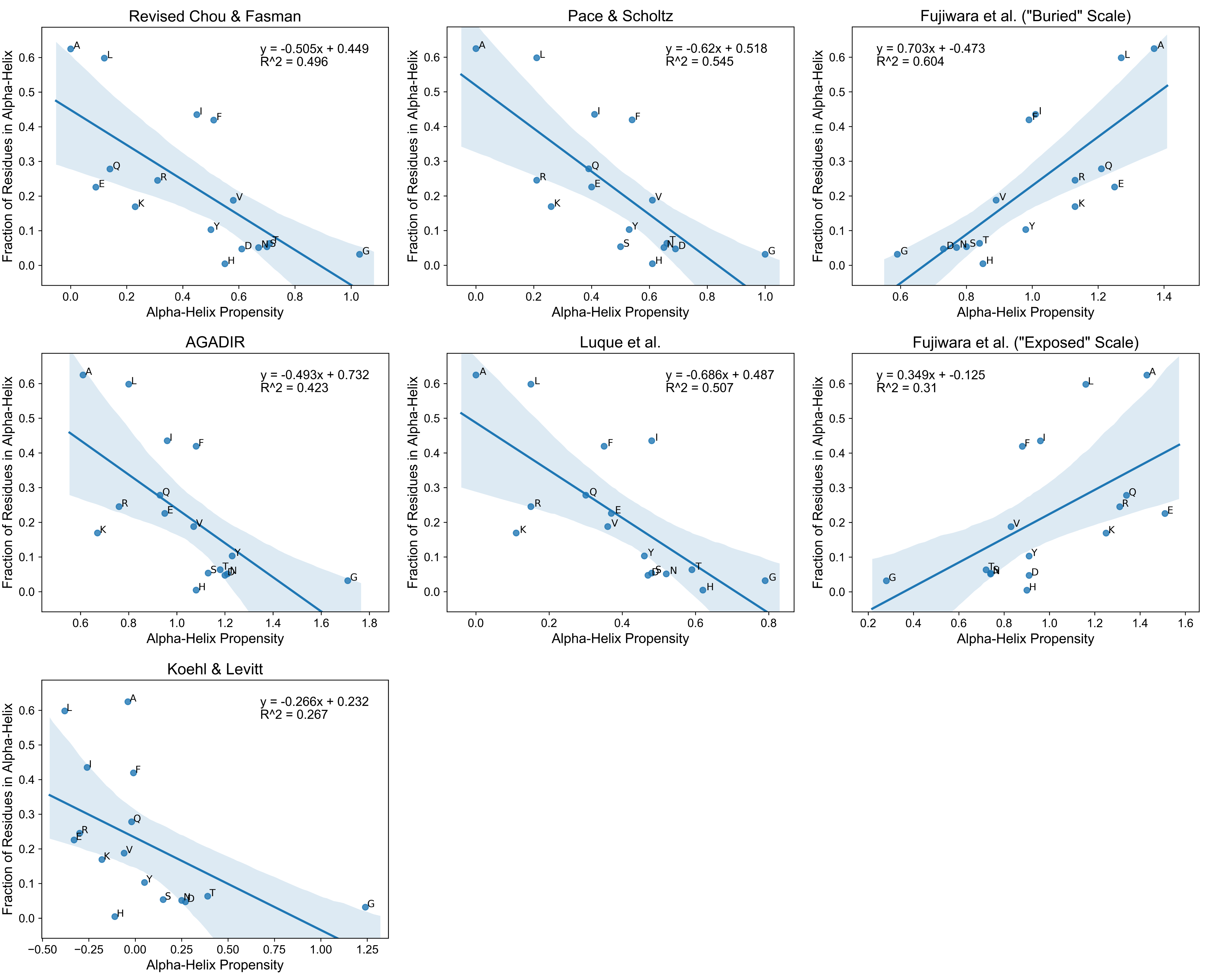

Supplement: S4 Fig — Scatter plots indicate all pairwise comparisons between the fraction of the LCD-defining residue in α-helices among each type of highly-enriched LCD and values from established α-helix propensity scales. Each shaded band indicates the 95% confidence interval around the regression line. (TIF) [file pcbi.1007487.s005.tif]

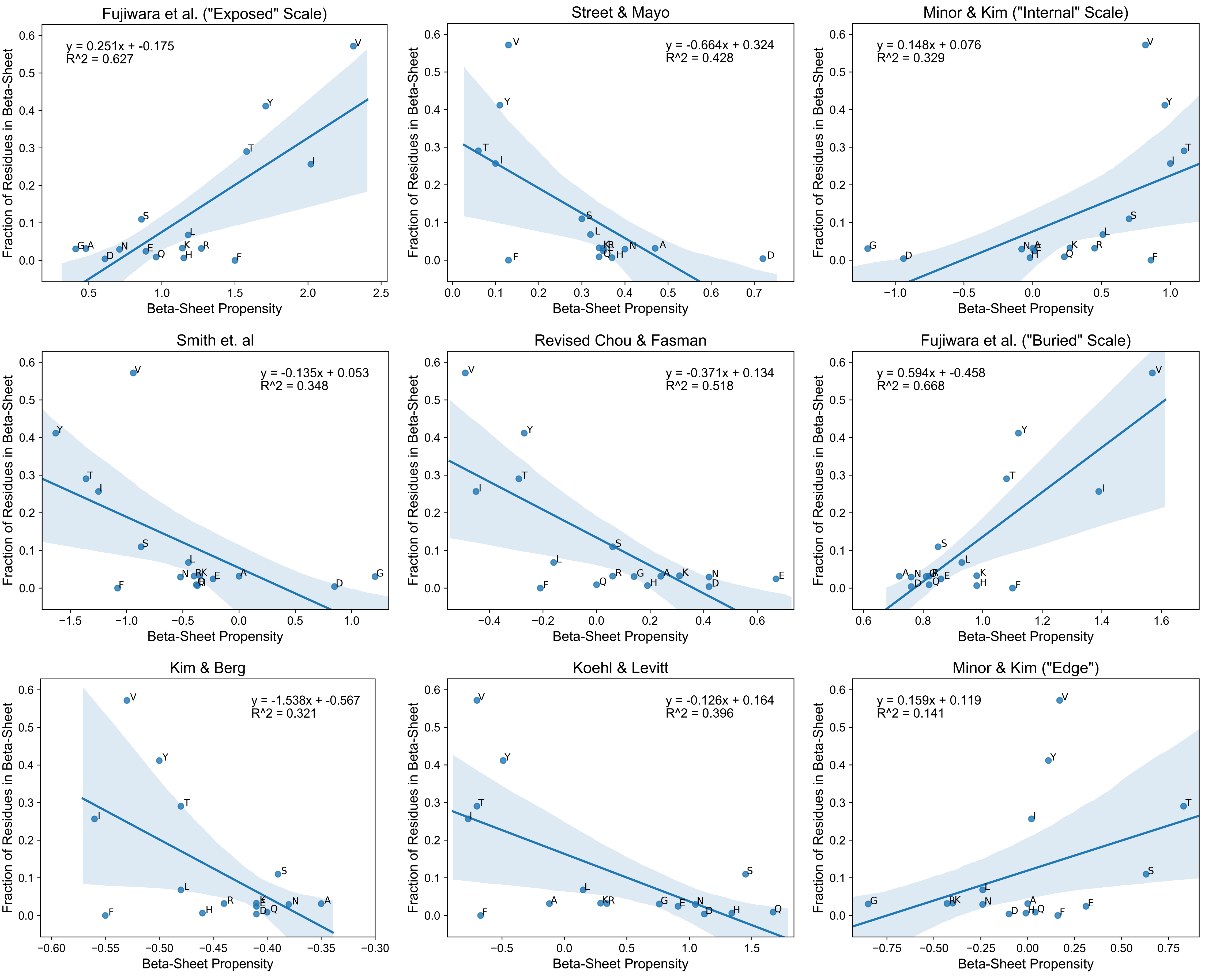

Supplement: S5 Fig — Scatter plots indicate all pairwise comparisons between the fraction of the LCD-defining residue in β-sheets among each type of highly-enriched LCD and values from established β-sheet propensity scales. Each shaded band indicates the 95% confidence interval around the regression line. (TIF) [file pcbi.1007487.s006.tif]

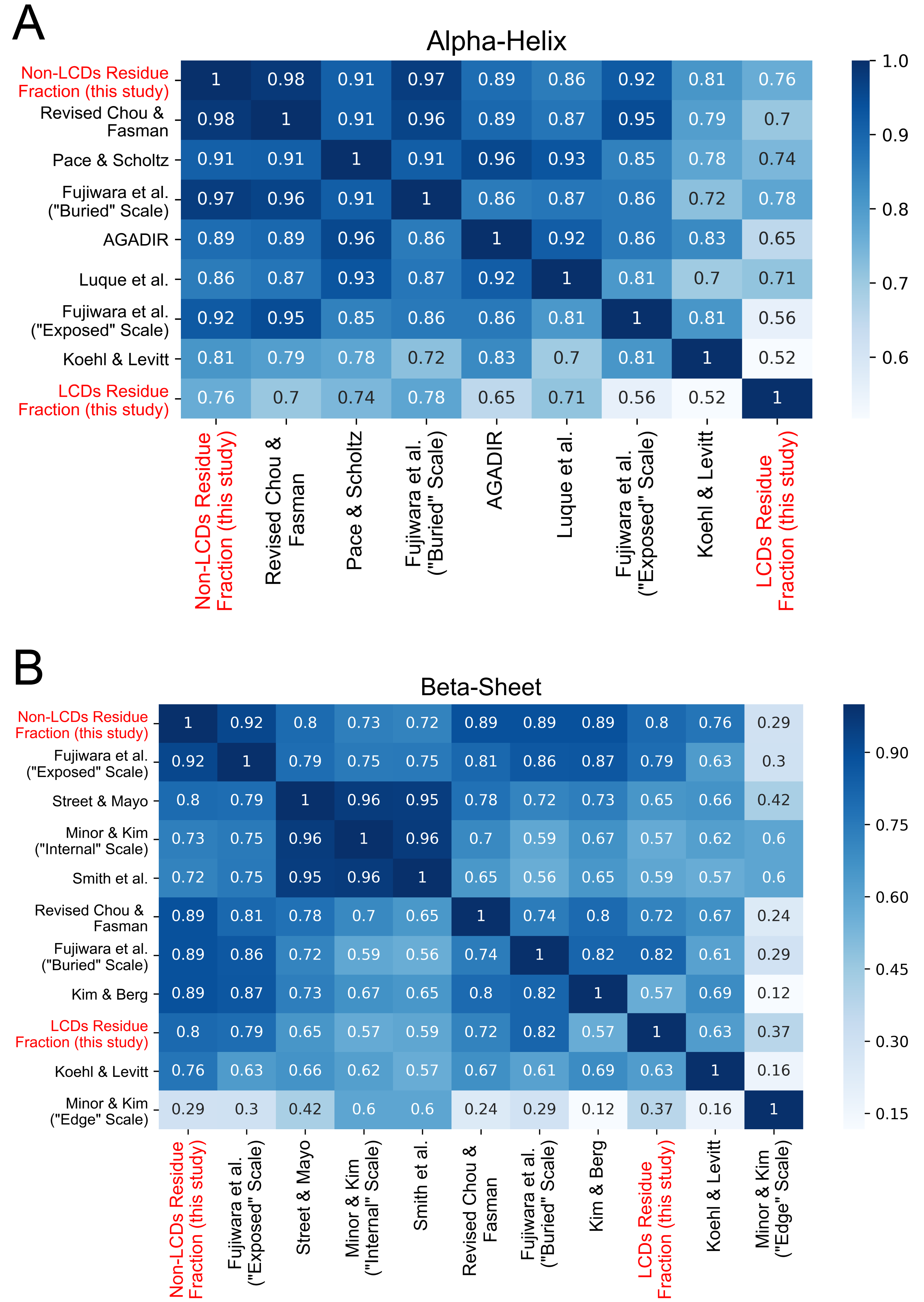

Supplement: S6 Fig — All pairwise comparisons between secondary structure propensity scales, as well as the fraction of each amino acid corresponding to each secondary structure type among highly-enriched LCDs and non-LCD regions, were performed for α-helix (A) and β-sheet (B) propensity scales (see also S4 and S5 Figs for each pairwise correlation plot). Heatmap intensities and corresponding values indicate the absolute value of the Pearson correlation coefficient for each comparison. To indicate general ranking with respect to the overall degree of correlation with all other α-helix or β-sheet propensity scales, the heatmaps were pre-sorted based on the average absolute correlation coefficient in descending order (top-to-bottom, and left-to-right). (TIF) [file pcbi.1007487.s007.tif]

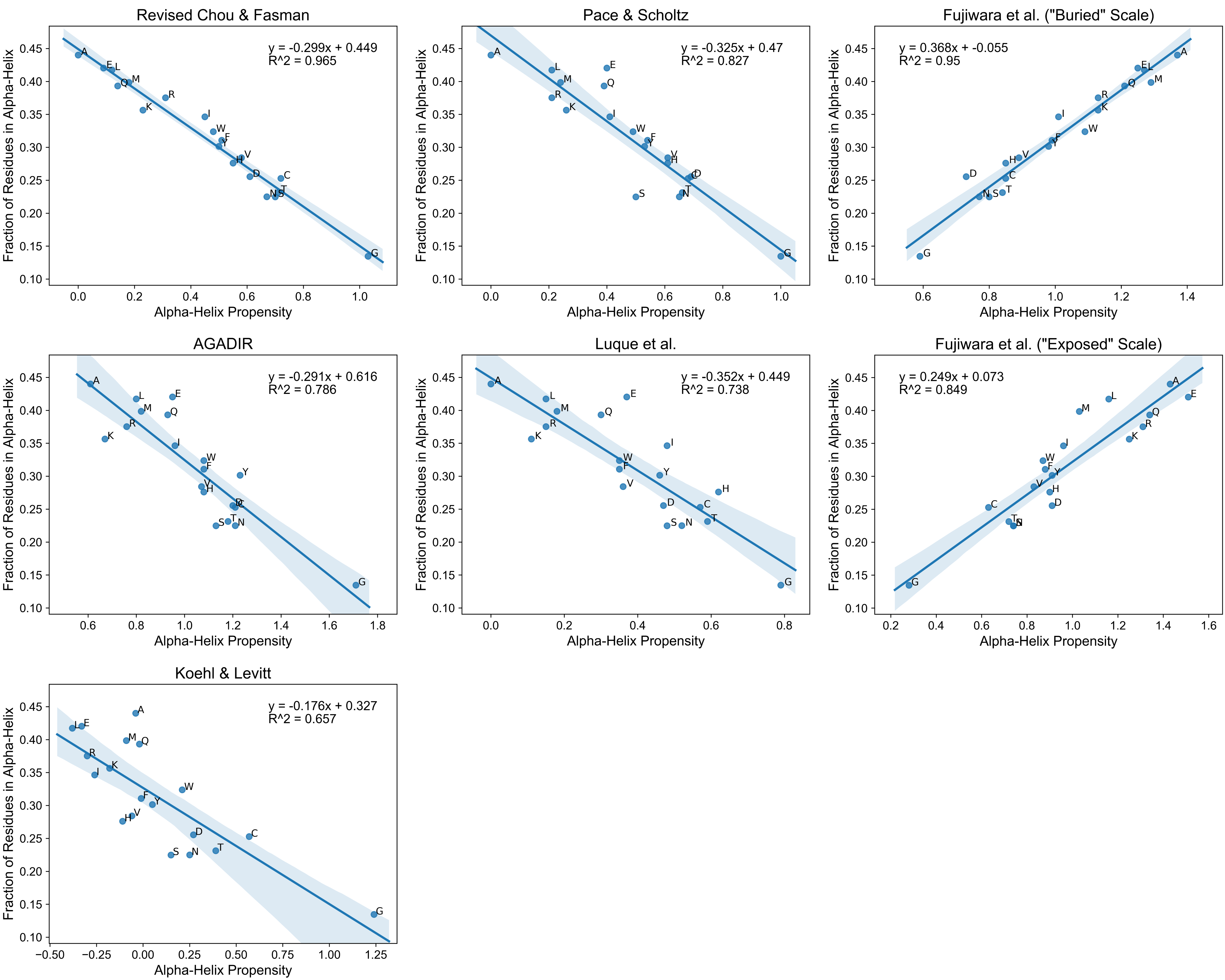

Supplement: S7 Fig — Scatter plots indicate all pairwise comparisons between the fraction of each residue in α-helices among the complementary set of non-LCD regions for each class of LCD and values from established α-helix propensity scales. Each shaded band indicates the 95% confidence interval around the regression line. (TIF) [file pcbi.1007487.s008.tif]

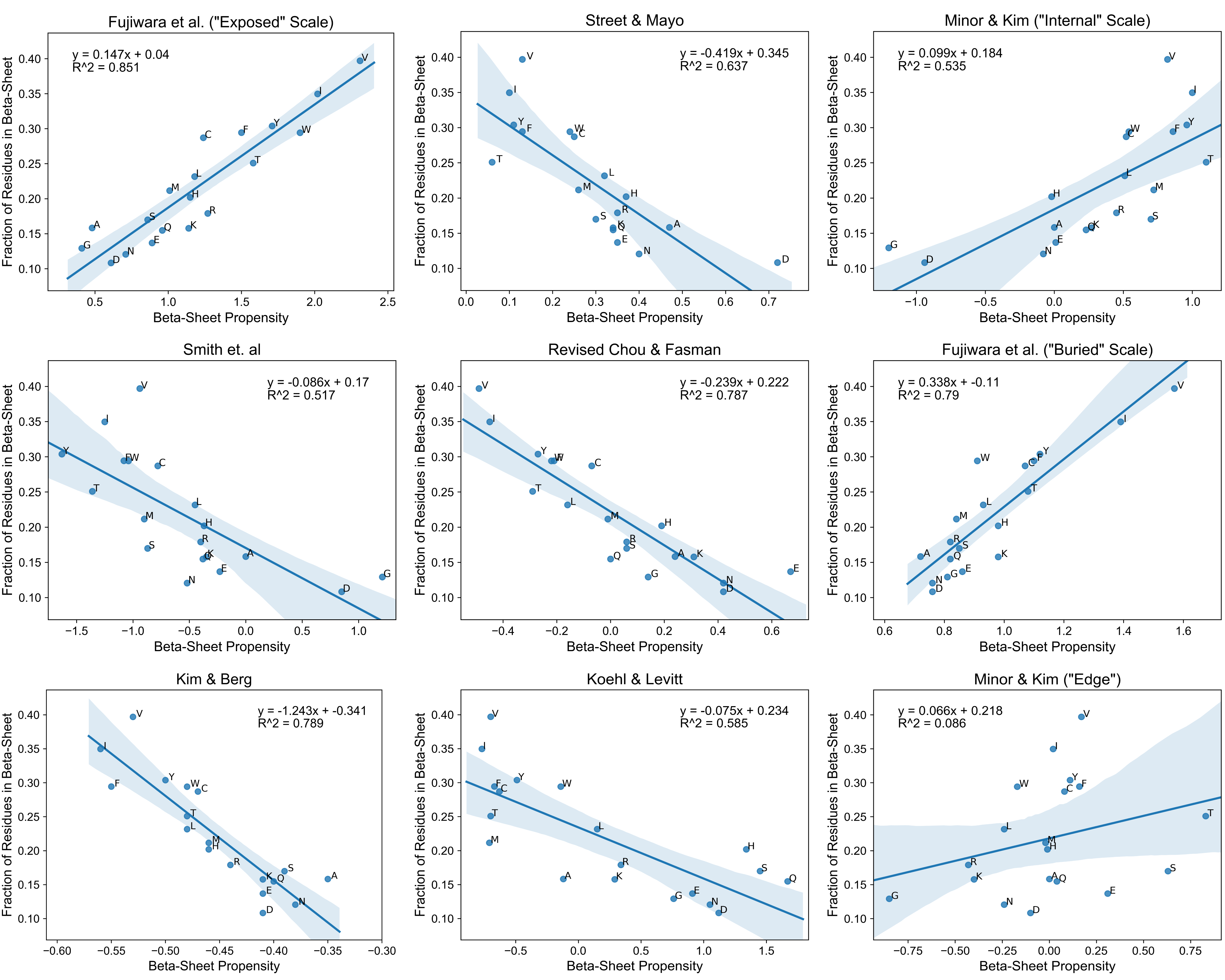

Supplement: S8 Fig — Scatter plots indicate all pairwise comparisons between the fraction of each residue in β-sheets among the complementary set of non-LCD regions for each class of LCD and values from established β-sheet propensity scales. Each shaded band indicates the 95% confidence interval around the regression line. (TIF) [file pcbi.1007487.s009.tif]

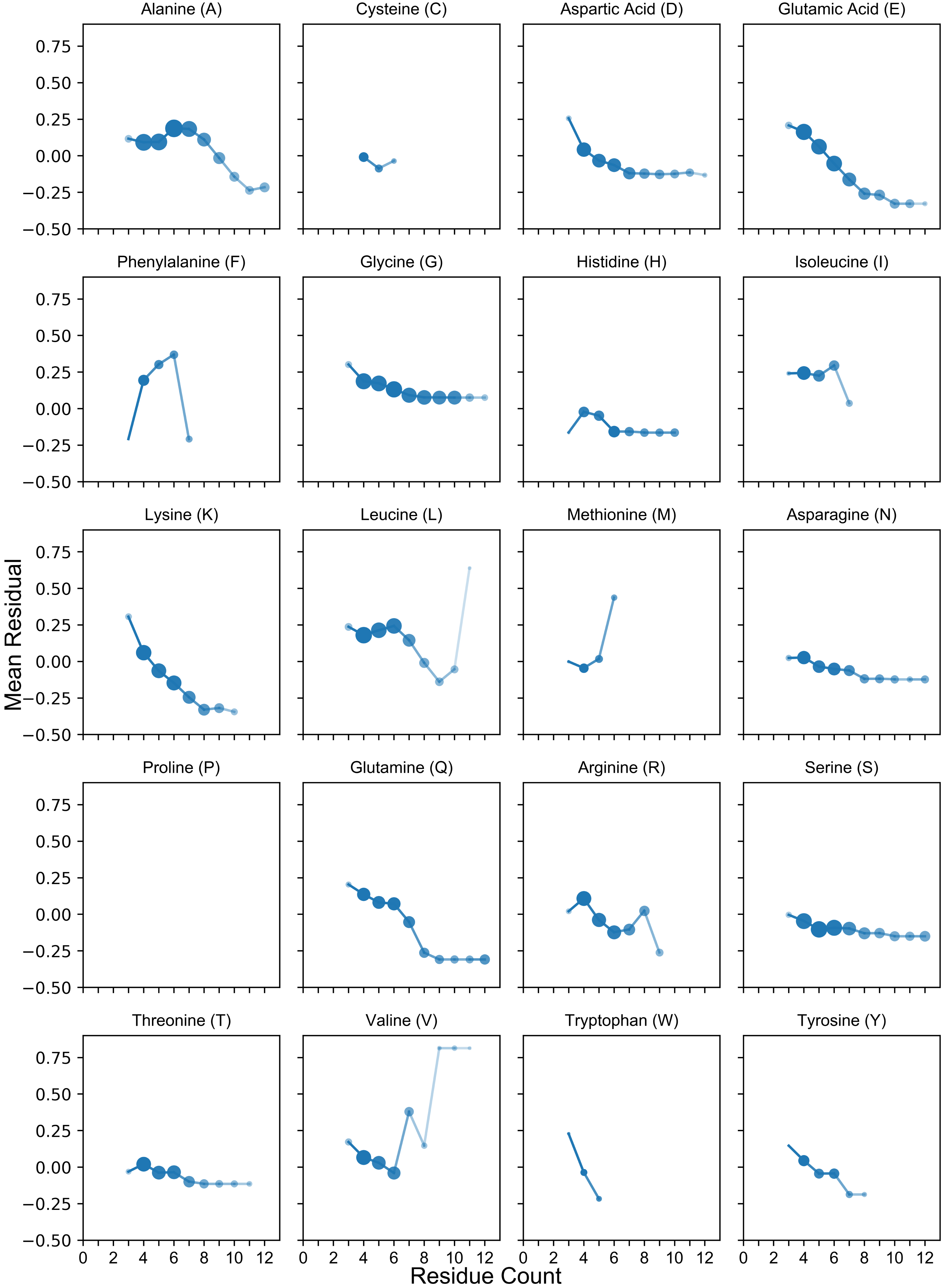

Supplement: S9 Fig — For each residue count bin, the fraction of the LCD-defining amino acid in α-helices was calculated separately for each LCD class. Pairwise regression analyses were performed between each α-helix propensity scale and the fraction of α-helical residues among highly-enriched LCDs. From the regression analyses, the residual values for each LCD class (i.e. each amino acid) were averaged across all α-helix propensity scales. This process was repeated independently for each residue count bin, and the resulting mean residual values are indicated in the figure above. Additionally, the size of each point (as well as the opacity of each point and the opacity of the preceding line segment) reflects the sample size for each residue count bin for each LCD class (i.e. the number of protein regions that were parsed into each bin based on the composition of the amino acid indicated in each subplot title). (TIF) [file pcbi.1007487.s010.tif]

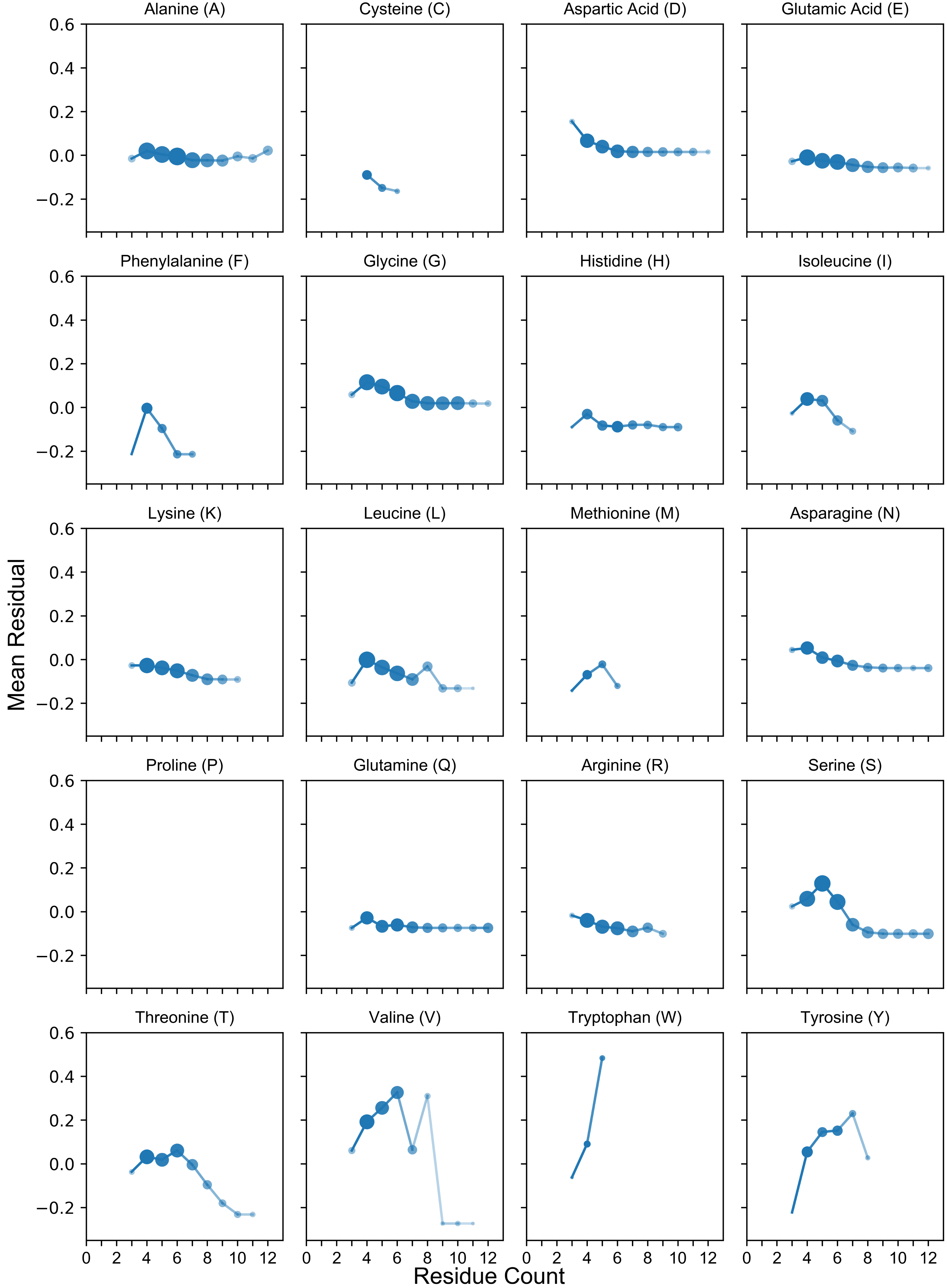

Supplement: S10 Fig — Mean residual values were calculated from pairwise regression analyses between observed fraction of LCD-defining residues in β-sheets among highly-enriched LCDs and each of the β-sheet propensity scales. Regression analyses, calculation of the residuals, and plotting were performed as indicated in the S9 Fig legend. (TIF) [file pcbi.1007487.s011.tif]

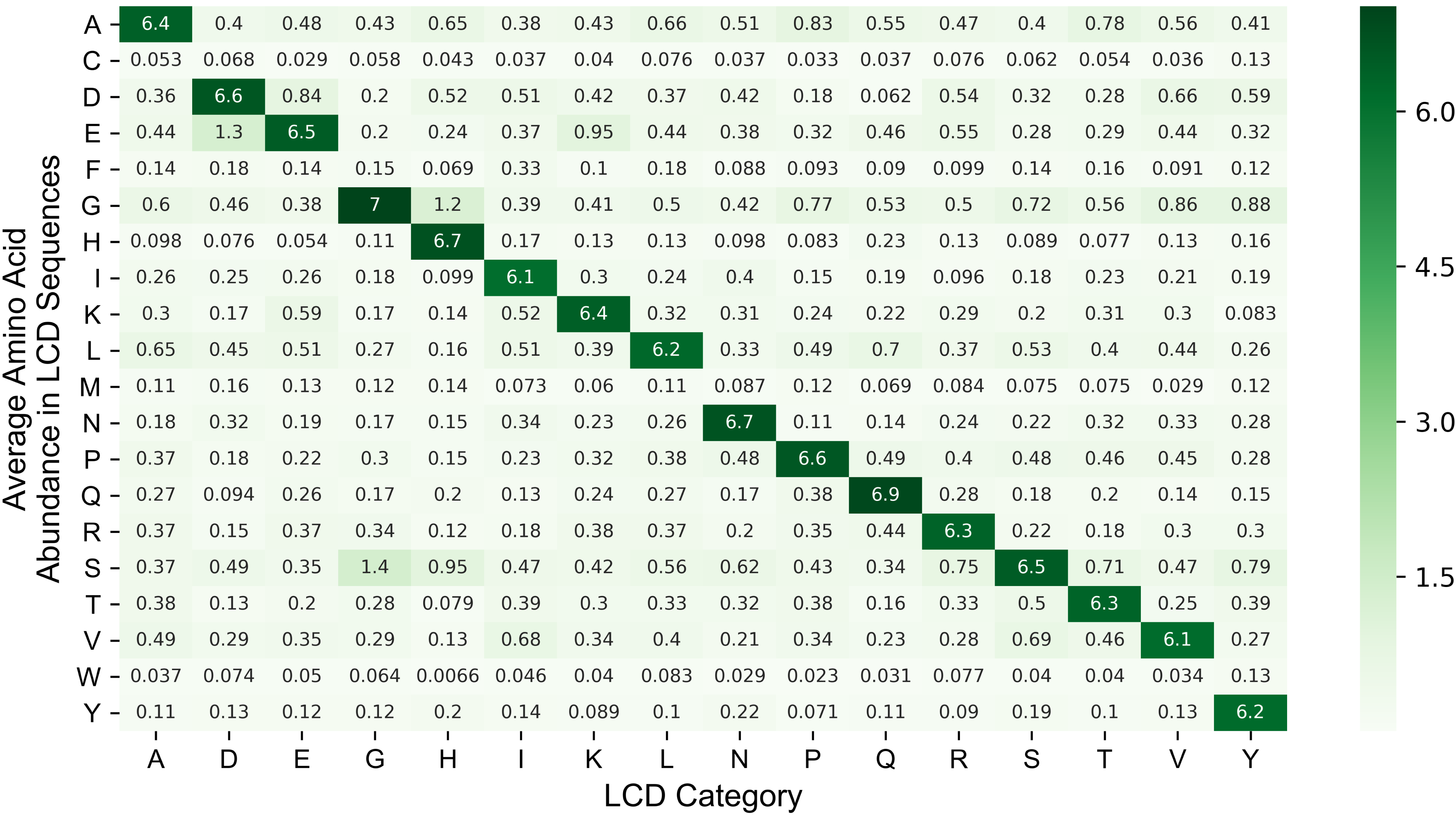

Supplement: S11 Fig — Heatmap indicating the average frequency of all residues calculated from the highly-enriched LCD sequences within each LCD category. (TIF) [file pcbi.1007487.s012.tif]

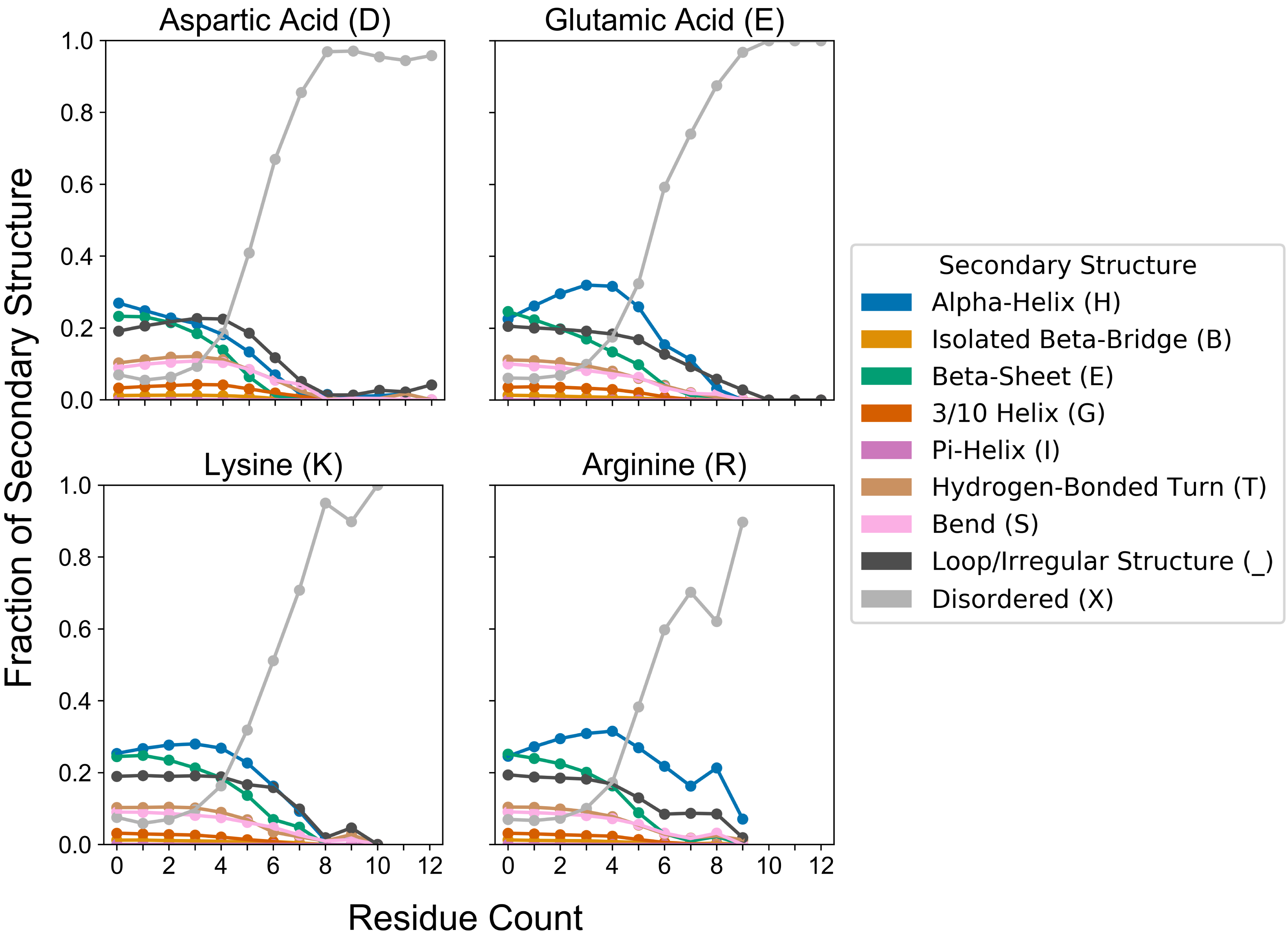

Supplement: S12 Fig — Secondary structure proportions were re-calculated for each of the main charged residues while eliminating all windows that contain an oppositely charged residue (see Methods). In all cases, the α-helix peaks exhibit a slight downward shift (compare with Fig 4), suggesting that charged single α-helices constitute only a minor contribution to the overall shape and magnitude of the α-helix curves for charged residues. (TIF) [file pcbi.1007487.s013.tif]
