# Supplementary material for: Atypical structural tendencies among low-complexity domains in the Protein Data Bank proteome
Source: PLoS Comput Biol. 2020 Jan 27;16(1):e1007487. doi: 10.1371/journal.pcbi.1007487 (PMC7004392; doi:10.1371/journal.pcbi.1007487)
Supplement: Attachment — Submitted filename: review.pdf [file pcbi.1007487.s019.pdf]

The authors have performed an extensive analysis of extant PDB data to examine low-complex domains within these structures. They have succinctly and clearly explained their methods and helped demonstrate that “low complexity domains” should not be considered synonymous with disordered domains. In additional to providing useful insight, the nature of the writing, analysis, and methods makes this an excellent example of a pedagogically inclined study that one might share with students for performing the type of analysis. Reaching a balance of sufficient but not overwhelming detail is challenging in this type of study, and I commend the authors for their effort here. I have a few minor points, but nothing substantial.

General points:

- It would be rather interesting to determine the relative enrichment of specific types of amino-acid enriched LCDs in the PDB vs. a given proteome. Specifically (as an example), normalizing for absolute number of residues scanned, to what extent do we see fewer G-rich LCDs in the PDB vs. the human (or yeast) proteome. This would be a useful analysis because my hunch is the PDB should be pretty depleted for disordered regions (given it’s a structural database…) so even though we see some G-rich LCDs in the PDB (4474, in fact) and some in the yeast proteome (538), GIVEN the relative differences in the size of those two databases is the PDB enriched or depleted for G-rich LCDs when compared to the yeast proteome? Assuming the PDB is reasonably representative in terms of amino acid composition from folded proteins *in general* this may help assess to what extent LCDs found in folded vs. disordered proteins.

- I assume just an issue with the PDF compression, but the figures were of rather low quality.

- In figure 4, where trends start to become non smooth and jagged, does this reflect the rapid drop off in the numbers of sequences? If yes, it may be useful to include a bar-chart axis above each panel showing the absolute number associated with each residue count. As someone who works in this field it’s clear this is probably happening (i.e. as you get to residue counts of 8+ there are maybe only a handful of sequences) but for someone unaccustomed to this they may read more into the shapes than is really warranted.

- Is the (by eye, anyway) correlated lysine/glutamate smooth peak from EK helices (such as those found in myosin). If yes, it might be useful to call this out, as these types of charged Single Alpha Helices (SAHs) are well characterized (see Süveges, D., Gáspári, Z., Tóth, G. & Nyitray, L. Charged single alpha-helix: a versatile protein structural motif. Proteins 74, 905–916 (2009).)

Book-keeping points

- Citation need: "*Similarly, while enrichment of most hydrophobic amino acids is associated with low protein abundance, low protein half-life, and low translation efficiency, progressive enrichment of alanine or valine is associated with complete opposite trends in protein metabolism*". (I think even if same citation as previous paragraph repeat again).

- Figure 2 – define what residues are in the groups (hydrophilic, hydrophobic etc.)

- Figure 5 – it would be useful for each stacked bar-chart to include the *n* associated with the underlying data. This does not alter any conclusions; I'd just like to know how many regions are associated with each LCD category

- The propensity for N to form beta sheets (vs. Q) has been explored independently in the context of aggregation previously, and agrees with the conclusions extracted here. I bring this up not as a criticism, but as a useful anecdote that authors could use to indicate how their analysis can directly inform on solution biophysics (Lu, X. & Murphy, R. M. Asparagine Repeat Peptides: Aggregation Kinetics and Comparison with Glutamine Repeats. *Biochemistry* **54,** 4784–4794 (2015), and Halfmann, R., Alberti, S., Krishnan, R., Lyle, N., O'Donnell, C. W., King, O. D., Berger, B., Pappu, R. V. & Lindquist, S. Opposing effects of glutamine and asparagine govern prion formation by intrinsically disordered proteins. Mol. Cell 43, 72–84 (2011).
