# Supplementary material for: Atypical structural tendencies among low-complexity domains in the Protein Data Bank proteome
Source: PLoS Comput Biol. 2020 Jan 27;16(1):e1007487. doi: 10.1371/journal.pcbi.1007487 (PMC7004392; doi:10.1371/journal.pcbi.1007487)
Supplement: Attachment — Submitted filename: PLOS Comp Biol - Response to Reviewers.docx [file pcbi.1007487.s020.docx]

Response to Reviewer Comments

*We would like to genuinely thank the reviewers for their time and thoughtful consideration of our manuscript. We’ve found the criticisms exceedingly helpful, and believe that the revisions and additional analyses that we have performed as a direct result have substantially improved the manuscript. Below is a point-by-point response to each of the reviewer suggestions, with our responses clearly indicated in red italic font.*

**Reviewer #1:**

This article presents some analysis of the relationship between 'low complexity' and Protein Data Bank structural preferences. This is an interesting work, although I have some points for revision.

* The data set of protein chains is constructed using a sequence identity threshold of 90% to remove redundancy. This is high. Usually, people would use something lower like 40% or 30%. Can the authors justify such a high threshold? They should show that the at least some of the results are not affected by such a high threshold.

*This is a valid point. Our initial worry was that choosing a stricter homology threshold could selectively bias against LCDs, since LCDs might statistically tend to boost sequence alignment scores even for less-related proteins. However, we have performed additional analyses on a dataset derived from a 40% homology threshold and the results are nearly identical to those in the original manuscript. Therefore, we have retained our original analyses in the manuscript, and have included additional supplementary figures with the data corresponding to the 40% non-redundant PDB dataset for comparison (Figs S2-S3 of the revised manuscript).*

* There is some discussion about the performance of secondary structure propensity scales for low complexity sequences (centred around Figs S1, S2, etc.). There does not seem to be a comparative discussion of the performance of propensity scales for non-LCD segments of protein structures as well. I think the authors should add this comparison and discuss the differences.

*This is also an extremely valuable point, and something that we grappled with in the initial preparation of the manuscript. The main problem is that the method of analysis that we were using (i.e. parsing LCDs into amino acid categories, calculating the fraction of each secondary structure type associated with that category, then performing regression analyses) is, by definition, not possible for non-LCD regions, since there is no clear way to parse non-LCD regions into amino acid categories in this manner.*

*Therefore, we devised a new approach to perform these analyses, but would like to emphasize up-front that this change had extremely minor effects on the data and did not, in any way, change the fundamental conclusions of the paper. Briefly, in order to obtain secondary structure propensity values for each amino acid among non-LCD regions, we quantified the fraction of residues for each amino acid that were annotated as α-helix or β-sheet among non-LCDs (a full description of the method is described in the revised manuscript, and a new visual representation of the computational workflow is depicted in Fig 6 of the revised manuscript). To ensure a fair comparison, the same method was performed for each of the LCD categories. All of the regression analyses were then re-calculated using this new statistic. This affects Fig 7, Figs S4-S6, and Figs S9-S10 (numbering based on the revised manuscript). However, we again emphasize that this change effectively captures the same information described in the original manuscript and produces very similar results, yet allows us to directly compare the performance of secondary structure propensity scales on LCD regions vs. non-LCD regions. We encourage the reviewers to compare Fig 6 and Figs S1-S5 of the old manuscript with Fig 7, Figs S4-S6, and Figs S9-S10 of the revised manuscript, respectively. Importantly, complementary regression analyses with non-LCD regions yielded remarkably high correlations and low residual values, indicating that the existing secondary structure propensity scales accurately reflect structural tendencies among high-complexity sequences. As the reviewer suggested, these new data constitute an important benchmark for comparison, and they are now included in Fig 7 and Figs S6-S8.*

**Reviewer #2:**

The authors have performed an extensive analysis of extant PDB data to examine low-complexity domains within these structures. They have succinctly and clearly explained their methods and

helped demonstrate that “low complexity domains” should not be considered synonymous with

disordered domains. In additional to providing useful insight, the nature of the writing, analysis,

and methods makes this an excellent example of a pedagogically inclined study that one might

share with students for performing the type of analysis. Reaching a balance of sufficient but not

overwhelming detail is challenging in this type of study, and I commend the authors for their

effort here. I have a few minor points, but nothing substantial.

*We appreciate the reviewer’s positive feedback. We did indeed find it extremely challenging to balance concise descriptions with sufficient detail for readers to understand exactly how analyses were performed. We have kept this in mind while revising the manuscript, and hope that the revisions maintain this standard. Consequently, though we had to change the computational approach for the regression analyses pertaining to Fig 7, Figs S4-S10 (see response to Reviewer #1 critiques above), we have included a new cartoon (Fig 6) depicting the computational workflow for deriving the values used in the regressions.*

General points:

• It would be rather interesting to determine the relative enrichment of specific types of

amino-acid enriched LCDs in the PDB vs. a given proteome. Specifically (as an

example), normalizing for absolute number of residues scanned, to what extent do we

see fewer G-rich LCDs in the PDB vs. the human (or yeast) proteome. This would be a

useful analysis because my hunch is the PDB should be pretty depleted for disordered

regions (given it’s a structural database…) so even though we see some G-rich LCDs in

the PDB (4474, in fact) and some in the yeast proteome (538), GIVEN the relative

differences in the size of those two databases is the PDB enriched or depleted for G-rich

LCDs when compared to the yeast proteome? Assuming the PDB is reasonably

representative in terms of amino acid composition from folded proteins *in general* this

may help assess to what extent LCDs found in folded vs. disordered proteins.

*This is an important point. When interpreting Fig 2, we were focused mostly on the percentages of proteins with LCDs across each of the proteomes: however, many readers might focus on the raw frequencies, which could be mis-leading if they do not also take into account the relative sizes of the proteomes. We have included an additional panel in Fig 2 addressing this.*

*One of the caveats when making comparisons between the PDB proteome and other proteomes is that the PDB represents an amalgamation of proteins from different organisms: proteins from certain organisms (e.g. homo sapiens) are selectively studied, leading to their disproportionate representation within the PDB. This likely affects the type of relative comparison that the reviewer is alluding to. Therefore, in order to incorporate this relative comparison, while accounting for selection bias in the PDB, we examined the relative comparisons between the human proteome and the human PDB proteome (i.e. only PDB proteins that are human proteins), and between the yeast proteome and the yeast PDB proteome. Specifically, within each proteome, we calculated the fraction of residues within LCDs of that residue type for each of the amino acids. For instance, the fraction of alanine residues residing within alanine-rich LCDs was calculated for the yeast proteome. This process was repeated independently for all amino acids and all proteomes included in our study, and the results are now in Fig 2E.*

*Overall, we agree that this better highlights relative differences between the proteomes, and facilitates a more reader-friendly comparison of the proteomes since all of the data can be expressed on the same graph with the same axes (the differences in the required y-axis limits for panels A-D necessitated individual graphs for those data).*

• I assume just an issue with the PDF compression, but the figures were of rather low

quality.

*Thank you for pointing this out. However, we do believe that this issue was introduced when the PDF was generated. All of the figure files that we submitted with the manuscript were initially made at high resolution (600dpi) and extremely large, then shrunk to fit the desired dimensions. Therefore, the actual figure resolutions should be at or near the maximum resolution limits for the journal.*

• In figure 4, where trends start to become non smooth and jagged, does this reflect the

rapid drop off in the numbers of sequences? If yes, it may be useful to include a barchart

axis above each panel showing the absolute number associated with each residue

count. As someone who works in this field it’s clear this is probably happening (i.e. as

you get to residue counts of 8+ there are maybe only a handful of sequences) but for

someone unaccustomed to this they may read more into the shapes than is really

warranted.

*This is an excellent suggestion: we have included a new supplementary figure indicating the sample sizes in each bin. Fig 4 is already quite large yet the subplots for each amino acid are fairly small, and the y-axis indicating sample sizes would have to be compressed substantially to fit the data above each subplot. Therefore, we chose to include these sample size data as a separate supplementary figure but in the same format and with the same sizing. Our hope is that this will facilitate direct reader comparison/alignment of the two sets of graphs, and should work both for printed versions of the manuscript as well as digital copies.*

• Is the (by eye, anyway) correlated lysine/glutamate smooth peak from EK helices (such

as those found in myosin). If yes, it might be useful to call this out, as these types of

charged Single Alpha Helices (SAHs) are well characterized (see Süveges, D., Gáspári,

Z., Tóth, G. & Nyitray, L. Charged single alpha-helix: a versatile protein structural motif.

Proteins 74, 905–916 (2009).)

*This is an interesting point. We have included additional analyses which suggest that charged single alpha helices (CSAHs) may indeed contribute to the alpha helix peaks observed for the main charged residues, but are not the dominating or defining contribution.*

*To examine this effect for each of the charged residues independently, we scanned the PDB proteome to determine the secondary structure profiles as a function of composition as performed for the analyses in Fig 4: however, all windows containing one or more oppositely charged residue were discarded during analysis. For example, when determining the secondary structures as a function of E composition, any windows containing R or K were discarded altogether, thereby precluding the possibility of having patterned opposite charges characteristic of CSAHs in the same window. For all of the charged residues, the alpha helix peak is dampened slightly (∆=~0.02-0.08), but the overall alpha helix content and characteristic shapes of the curves are maintained (compare Fig 4 with Fig S12).*

*We have included a short discussion of these data in the Discussion section to highlight one of the broader challenges we consistently grapple with in bioinformatic studies of this scale: there is generally so much data that any number of specific examples can lead to interesting insights if explored in greater detail (which, in our view, constitutes one of the primary purposes of such broad bioinformatic analyses). Our initial study lays a necessary foundation by focusing on the structures associated with compositional enrichment for each of the amino acids separately. Once the foundation is set, follow-up studies (by us or others) can then focus on specific aspects of the trends we have observed, and CSAHs provide an interesting and concise example of this – we thank the reviewer for this suggestion.*

Book-keeping points

• Citation need: “*Similarly, while enrichment of most hydrophobic amino acids is*

*associated with low protein abundance, low protein half-life, and low translation*

*efficiency, progressive enrichment of alanine or valine is associated with complete*

*opposite trends in protein metabolism*”. (I think even if same citation as previous

paragraph repeat again).

*Thank you - we have included this citation.*

• Figure 2 – define what residues are in the groups (hydrophilic, hydrophobic etc.)

*In order to maintain an adequate font size in the inset figures, the definitions of the amino acid groups have been included in the figure legend corresponding to Fig 2.*

• Figure 5 – it would be useful for each stacked bar-chart to include the *n* associated with

the underlying data. This does not alter any conclusions; I’d just like to know how many

regions are associated with each LCD category

*We agree, and have incorporated the sample size associated with each category for all stacked bar charts.*

• The propensity for N to form beta sheets (vs. Q) has been explored independently in the

context of aggregation previously, and agrees with the conclusions extracted here. I

bring this up not as a criticism, but as a useful anecdote that authors could use to

indicate how their analysis can directly inform on solution biophysics (Lu, X. & Murphy,

R. M. Asparagine Repeat Peptides: Aggregation Kinetics and Comparison with

Glutamine Repeats. *Biochemistry* **54,** 4784–4794 (2015), and Halfmann, R., Alberti, S.,

Krishnan, R., Lyle, N., O’Donnell, C. W., King, O. D., Berger, B., Pappu, R. V. &

Lindquist, S. Opposing effects of glutamine and asparagine govern prion formation by

intrinsically disordered proteins. Mol. Cell 43, 72–84 (2011).

*This is an excellent suggestion: we have included an additional point in the Results section highlighting this corroborative evidence.*
